# Reply to “Convergent and divergent selection in sympatry drive plumage evolution in woodpeckers”

**DOI:** 10.1038/s41467-019-14007-2

**Published:** 2020-01-09

**Authors:** Eliot T. Miller, Gavin M. Leighton, Benjamin G. Freeman, Alexander C. Lees, Russell A. Ligon

**Affiliations:** 1 Cornell Lab of Ornithology, 159 Sapsucker Woods Rd., Ithaca, NY 14850 USA; 2 Department of Biology, SUNY Buffalo State College, Buffalo, NY 14213 USA; 3 Department of Zoology, University of British Columbia, #4200-6270 University Blvd, Vancouver, BC V6T1Z4 Canada; 4 School of Science and the Environment, Manchester Metropolitan University, Manchester, UK

**Keywords:** Coevolution, Mimicry, Phylogenetics

**Replying to** G. Grether. *Nature Communications* 10.1038/s41467-019-14006-3 (2019)

We recently presented results showing that climate, habitat, and range overlap all influence the evolution of plumage coloration and patterning in a large clade of birds, the woodpeckers^1^. Of particular significance, we found that after accounting for shared climate, habitat, and evolutionary history, species pairs with the most similar plumages showed a pronounced tendency to occur in sympatry. In a concurrent comment to our own here, Grether recognized this finding, but stated that we did not sufficiently highlight “evidence for accelerated divergence in sympatry,” and hence did not detect signals of both divergence and convergence in a large clade of animals for the first time. We herein respond to these comments.

In comparison to the large body of literature supporting character divergence in sympatry between close relatives (frequently ecological competitors), evidence in support of character convergence is much smaller. Darwin’s predictions of character divergence facilitating population persistence have largely set the research agenda on interspecific species interactions for the last 160 years^2,3^. Yet, under certain circumstances, species may evolve to closely resemble one another in sympatry. The reasons for this are varied, but of particular relevance to our recent paper^1^ is the theory of competitive mimicry^4^. In comparison to the many described cases of character divergence in sympatry^2^, fewer examples of character convergence caused by competitive mimicry have been described. These include situations where a mimic is able to fool either the model itself (convergent agonistic character displacement^5^ e.g., in fish^6^), or third parties^7–9^, and thereby gain access to contested resources. This dearth of well-demonstrated examples of competitive mimicry is especially true of birds, where despite over 150 years of speculation on what drives qualitatively identified purported plumage mimics^7,10–15^, few papers have tested whether these cases truly represent mimicry, or whether they might simply be adaptation to shared habitats or a product of shared evolutionary history^9^. Our study provided some of the first quantitative evidence that plumage mimicry occurs in birds and cannot be explained by other factors.

The graphical summary of woodpecker plumage mimicry in close sympatry was presented in Fig. 4 of our recent manuscript^1^, within which is an intriguing finding highlighted by Grether. While Fig. 4 highlights a significant positive association between species range overlap and close plumage similarity, there is also a strong and significant negative association between species range overlap and intermediate plumage similarity. Given the aforementioned robust body of literature on plumage divergence between close competitors in partial sympatry or parapatry, this result intrigued both us and Grether. Our caution in discussing this result in the original manuscript stemmed largely from the fact that plumage divergence was not an a priori focus of the study. Herein we discuss this pattern and whether it might be driven by divergent character displacement.

The key to understanding the modified Mantel correlogram in Fig. 4 is to consider that each point in the figure represents the correlation of a given set of pairwise comparisons of plumage dissimilarity with range dissimilarity. The data point in question here concerns species with plumage dissimilarities of 0.2–0.3, such as the illustrated difference between *Dryobates pubescens* and its near-parapatric congener *D*. *scalaris*. This data point illustrates that, when setting species pairs in this bin (“intermediately dissimilar pairs”) to zero and all other species pairs to one, there is a negative correlation between range and plumage dissimilarities, such that sympatry is associated with plumage divergence, whereas allopatry is associated with plumage convergence. This overall negative correlation strongly supports the divergence in sympatry argument made by Grether, and thus his point is well made. That said, when we used our new method of identifying high-leverage, intermediately dissimilar pairs, we discovered that there may be alternative explanations for some of these divergence examples. As an example of what appears to be a classic case of plumage divergence in partial sympatry, one of the sympatric pairs with the highest leverage was *Dendrocopos himalayensis* and *Dendrocoptes auriceps*, two presumed ecological competitors that replace one another elevationally in the Himalayas^16^ (Fig. 1a, b). Yet, another species pair with high leverage was *Reinwardtipicus validus* and *Hemicircus concretus* (Fig. 1c, d), the former of which weighs approximately 5.7 times that of the latter^16^; there is presumably little ecological interaction between these species. Indeed, the divergence between these latter two species may be driven in part by notable convergence between *H*. *concretus* and [a third sympatric species] *Meiglyptes tristis* (Fig. 1e). In short, while we think Grether is likely correct in identifying this overall trend as supporting divergent agonistic character displacement, we urge caution in this interpretation as some examples do not strongly support this conclusion, and our ability to disentangle these opposing processes is currently methodologically limited. Distance-based, multivariate phylogenetic analytical approaches are an area of active research, and future advances should allow these intricacies to be adequately teased apart.

**Fig. 1:**
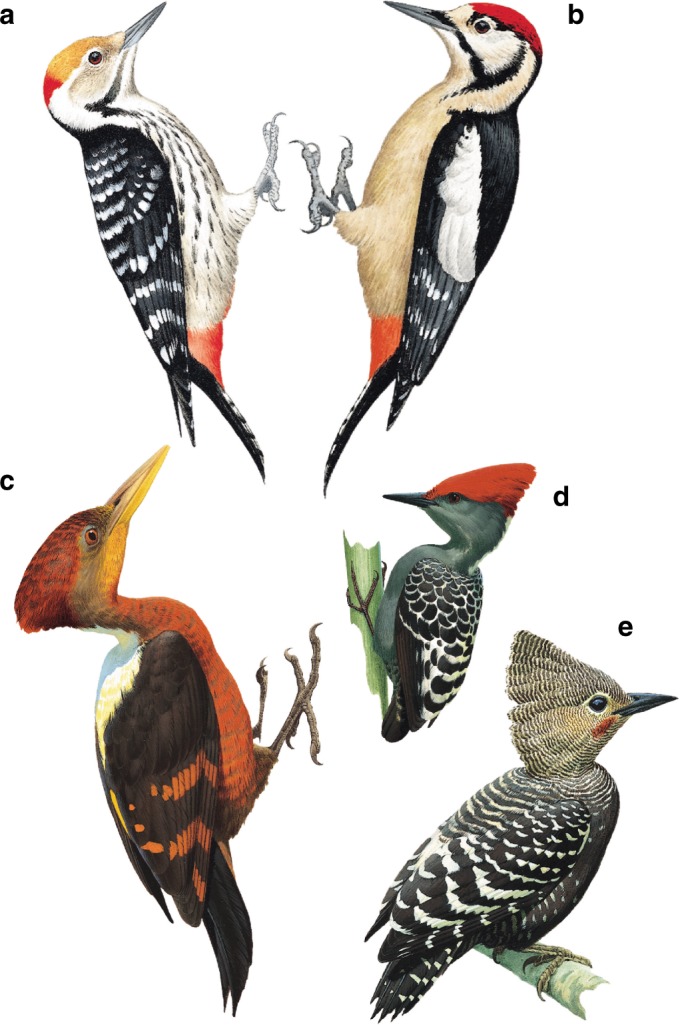
Two groups of sympatric woodpecker species whose plumage has been shaped by co-occurring heterospecifics. **a** *Dendrocoptes auriceps* and **b** *Dendrocopos himalayensis* replace one another elevationally in the Himalayas, and have diverged more than expected based on their shared evolutionary history, habitat, and climate. **c** *Reinwardtipicus validus* and **d** *Hemicircus concretus* are sympatric on the island of Java, and likewise have diverged notably in plumage. However, (**d**) and *Meiglyptes tristis* (**e**) have converged on one another in sympatry in Java; our ability to analytically disentangle these competing patterns is currently limited. Illustrations © HBW Alive/Lynx Edicions.

We conclude with three additional points that merit further discussion. First, with regards to terminology, while many cases of divergence might be good examples of divergent agonistic character displacement, the only empirically supported hypothesis of what might drive the instances of mimicry in woodpeckers is not convergent agonistic character displacement (mimicry intended to fool the model). Rather, this appears to be mimicry intended to fool third-parties (type-D disjunct defensive antergic mimicry)^4^. Second, in our original manuscript, we referred to the unlikely possibility that “allopatry in and of itself drives plumage divergence between somewhat similar looking species pairs” (=intermediately dissimilar pairs)^1^. But, as noted above, the overall correlation was such that allopatric intermediately dissimilar species pairs tend to be convergent, not divergent, in plumage. Thus, the original manuscript should have said it was unlikely that allopatry drives plumage convergence. Our point remains the same—the correlation discussed in our response here is likely driven by selection exerted by sympatric species, since allopatric species pairs cannot exert direct selection pressures on one another (caveats about historical range shifts notwithstanding). Finally, although we think caution is warranted when assessing the body-region-specific results discussed in our original manuscript, since these are unlikely to be completely independently evolving plumage modules, we found it notable that our results in support of competitive mimicry in woodpeckers were strongest when assessed at the level of back and tail plumage—those parts of the bird most exposed to heterospecific receivers. In contrast, belly plumages in woodpeckers were better predicted by a Brownian motion model of evolution, and range overlap was not significantly associated with belly plumage evolution. We think future work teasing apart these body-region-specific patterns in both woodpeckers and other taxa will be especially fruitful.

## Data Availability

All data supporting the findings of this study are available within the original paper and its supplementary information files.
